# Living in a Larger Body: Do Exercise Motives Influence Associations between Body Image and Exercise Avoidance Motivation?

**DOI:** 10.3390/ijerph18010072

**Published:** 2020-12-24

**Authors:** Christy Greenleaf, Alexandra M. Rodriguez

**Affiliations:** Department of Kinesiology, University of Wisconsin Milwaukee, Milwaukee, WI 53211, USA; rodri679@uwm.edu

**Keywords:** body image, exercise, motivation, empowerment

## Abstract

The study explored reasons for exercise as possible mediators of the relationship between body appreciation and exercise avoidance motivation and between body surveillance and exercise avoidance motivation. Using a cross-sectional design, 131 women with a body mass index (BMI) of 25 or higher completed measures of body surveillance, body appreciation, reasons for exercise, and exercise avoidance motivation. Mediation analyses indicated that appearance-oriented reasons for exercise partially mediated the body surveillance–exercise avoidance motivation relationship. Health and fitness professionals, organizations, and environments should avoid reinforcing appearance-oriented reasons for exercise. Rather, empowering exercise experiences and environments should be created as they seem to benefit women regardless of reasons for exercise.

## 1. Introduction

Exercise environments may be unwelcoming for women whose bodies deviate from size-normative standards of attractiveness, beauty, and the thin ideal [1,2]. Indeed, women living in larger bodies report a desire to avoid physical activity and exercise [3] and face many barriers to physical activity (e.g., apparel, stigma) [4,5,6]. Yet, not all actually avoid exercise [2,7,8]. How women feel about their bodies and their exercise motives may play a role. Reasons for exercising, often health- and appearance-oriented, seem to be associated with different experiences in body image and psychological health. Among women living in larger bodies, exercising for health-related reasons is related to higher levels of body satisfaction [9], whereas appearance-oriented reasons are associated with lower levels of body satisfaction, self-esteem, and body esteem [10,11,12]. This pattern of associations is not unique to women living in larger bodies [13]. Because women living in larger bodies are likely to encounter weight-related stigma and have reported the desire to avoid physical activity, we were curious about how physically active women experience their bodies in relationship to reasons for exercise and motivation to avoid exercise. 

As a first step, we hypothesized that physically active women living in larger bodies would experience some degree of motivation to avoid exercise. This hypothesis is justified as previous research has documented positive associations between body mass index (BMI) and motivation to avoid exercise [3]. We also expected that, in line with previous research that has explored exercise behavior [14], women’s feelings about their bodies would be associated with the extent to which they desire to avoid exercise. Specifically, higher levels of body surveillance, or monitoring one’s body appearance with concerns about how others are evaluating it, would be related to greater motivation to avoid exercise, whereas higher levels of body appreciation would be related to less motivation to avoid exercise. Finally, we hypothesized that women’s reasons for exercise would influence the relationships between body surveillance and exercise avoidance motivation and between body appreciation and exercise avoidance motivation. Below, we outline the theoretical and empirical rationale for our hypotheses.

To provide theoretical grounding for our work, we explored body surveillance, a key construct from Objectification Theory [15]. Objectification Theory posits that women are socialized to internalize a third-person perspective of their own body. Self-objectification can result in frequent and pervasive monitoring of one’s body, or body surveillance. Women who engage in body surveillance experience a host of negative psychological and behavioral outcomes including body shame, appearance anxiety, disturbed eating attitudes, and pathogenic weight control behaviors [16]. These outcomes are also commonly reported among women who experience stigma stemming from pervasive anti-fat bias [17]. Body surveillance is associated with stronger appearance-related motives [13,14], however, connections to exercise avoidance motivation have yet to be examined. Additionally, Homan and Tylka, who have done work in this area, did not report participants’ body mass index (BMI) or have screened out outliers with a high BMI [13,14], thus it is unknown how these associations might reflect the experiences of women living in larger bodies.

Given limited research on body surveillance and exercise avoidance motivation to date and the absence of women with larger bodies from samples, we wanted to explore how experiences of body surveillance are related to motivation to avoid exercise and how reasons for exercise, appearance- and health-oriented, might mediate that association among women living in larger bodies. In particular, we were curious about the extent to which appearance-oriented motives might strengthen associations between body surveillance and motivation to avoid exercise and health-oriented motives might weaken that association. Conceptually, it seems reasonable that women who are driven by appearance experience a stronger connection between body surveillance and exercise avoidance motivation. That is, women living in larger bodies who indicate a higher degree of monitoring and concern about their body appearance yet are also motivated by appearance may experience dissonance and have a stronger desire to avoid exercise. On the other hand, health motives for exercise might buffer or lessen the association between surveillance and avoidance motivation, thus acting as a possible protective mechanism. Because research in this area is relatively limited, we view our work as a preliminary step toward building a clearer picture of how women’s reasons for exercise interact with their experiences of body surveillance and desire to avoid exercise.

More recently, researchers have moved beyond studying negative aspects of body image, such as body surveillance, to focus on understanding positive feelings toward one’s body [18]; thus, we sought to build upon that approach by also exploring body appreciation. Body appreciation, unlike body surveillance, is associated with positive psychological and behavioral experiences, including self-compassion [19] and intuitive eating [20]. Associations of body appreciation with exercise motives are not well understood. In a study of college-age women, Homan and Tylka [14] found that body appreciation was not associated with appearance-oriented reasons for exercise; yet appearance motives moderated the association between frequency of exercise and body appreciation. Health and fitness motives were not assessed, and the BMI of participants was not reported. In 2015, Tylka and Homan [13] reported that both appearance and functional motives for exercise were associated with body appreciation; stronger appearance motives were negatively correlated to body appreciation and higher functional motives were positively associated with body appreciation.

The work by Homan and Tylka [13,14] provides us with an initial starting point; however, additional research is needed to better understand how reasons for exercise might play a role in associations between body appreciation and exercise avoidance motivation, particularly among women living in larger bodies. As such, we explored reasons for exercise as potential mediators of the relationship between body appreciation and exercise avoidance motivation. We were specifically interested in determining if appearance motives might lessen associations between body appreciation and motivation to avoid exercise as well as if health motives might increase the strength of association. We expect that body appreciation will be negatively associated with exercise avoidance motivation and we wonder if, for women who experience stronger appearance motives for exercise, that association is weaker than for women who have stronger health-related motives. Because body appreciation is associated with a myriad of positive health outcomes (e.g., higher self-esteem, intrinsic motivation) [19,21], motives that might offset or strengthen that benefit need to be understood and either limited or supported.

In this study, we sought to examine associations between reasons for exercise (appearance- and health-oriented), motivation to avoid exercise, body surveillance, and body appreciation, and explore reasons for exercise as possible mediators between body surveillance, body appreciation, and exercise avoidance motivation. To summarize, we tested the following hypotheses: 

Most physically active women living in larger bodies will experience some degree of motivation to avoid exercise,

- Body-related experiences, body surveillance and body appreciation, will be related to motivation to avoid exercise,
- Appearance-oriented reasons for exercise will mediate the association between body surveillance and exercise avoidance motivation, and
- Health-oriented reasons for exercise will mediate the association between body appreciation and exercise avoidance motivation.

Because of our interest in understanding the experiences of women living in larger bodies, we developed a purposeful sampling methodology, as outlined below.

## 2. Materials and Methods

### 2.1. Participants and Procedures

Participants were recruited through Qualtrics online sample panel services (https://www.qualtrics.com/research-services/online-sample/) as part of a larger study on weight bias internalization and embodiment. Eligible participants were aged 18 or older, identified as female, reported wearing clothing in a United States size 14 or larger [22], and engaged in purposeful physical activity at least 2 days a week. To increase the likelihood that participants were women living in larger bodies, only those with a body mass index (BMI) of 25 or higher were included in the sample. In total, 131 women (*M*_age_ = 57.23, *SD* = 15.70) were included, with most identifying as Caucasian (83.2%), African American (9.2%), multi-ethnicity (4.6%), Hispanic (2.3%), and Native American (0.7%).

This study was approved by the [blank for review] Institutional Review Board (IRB) (#18.145). Prior to beginning the survey, participants reviewed consent information; implied consent was given by navigating to the first page of the survey. Participants completed a series of quantitative measures that were presented in random order. Those who completed the surveys received compensation from Qualtrics.

### 2.2. Measurement

#### 2.2.1. Body Surveillance

The Body Surveillance subscale from the Objectified Body Consciousness Scale (BS-OBC) measures body self-monitoring or consciously attending to one’s body [23]. The subscale contains 8 items (e.g., “I think more about how my body feels than how my body looks”) rated using a 7-point scale (1 = *strongly disagree*, 7 = *strongly agree)*. All but two items are reverse scored, then averaged; lower scores indicate lower levels of body surveillance. In a sample of women, Overstreet, Quinn, and Agocha [24] reported an internal consistency of 0.89, which is consistent with the current study sample (α = 0.86). 

#### 2.2.2. Body Appreciation

The Body Appreciation Scale-2 (BAS-2) assesses the extent to which individuals hold favorable opinions toward, accept, and respect their own body [25]. A 5-point scale (1 = *never*, 5 = *always*) is used to indicate agreement with 10 statements (e.g., “I respect my body”). Scores for this scale are calculated by averaging the responses of all items, with higher scores reflecting greater body appreciation. Among a sample of women, O’Neill, Winter, and Pevehouse [26] found an internal consistency (α = 0.93) similar to that of the current sample (*α* = 0.96).

#### 2.2.3. Reasons for Exercise

Reasons for Exercise Inventory (REI) [27] assesses motives for engaging in exercise. Individuals rate the importance of each of 24 statements (e.g., “To improve my cardiovascular fitness”, “To alter a specific area of my body”) on a 7-point scale (1 = *not at all important*, 7 = *very important*). Similar to the approach taken by others [9,12], we used two categories: motives for improved appearance (8 items) and motives for health and fitness (8 items). Scores for each subscale are calculated by averaging the items that pertain to that subscale; higher scores indicate stronger endorsement of the reason for exercise. Vartanian et al. [12] reported internal consistencies for the appearance motives (α = 0.78) and health and fitness motives (α = 0.81) subscales. The internal consistencies for each subscale in the current study sample were appearance (α = 0.93) and health and fitness (α = 0.92).

#### 2.2.4. Exercise Avoidance Motivation

Exercise avoidance motivation is an individual’s desire to not engage in physical activity. The construct was measured via 3 items from the Exercise Avoidance Motivation scale (EAM) [28]. A 7-point scale (1 = *not at all true*, 7 = *completely true*) is used to rate the extent to which each statement is true (e.g., “I am too embarrassed to participate in physical activity in public places”). The average is taken as the scale score; higher scores reflect greater motivation to avoid physical activity. Vartanian and Novak [28] found an internal consistency of the original 8-item scale (α = 0.79) among a predominately female sample; in the current study sample, α = 0.88.

### 2.3. Planned Analyses

Using SPSS 25 (IBM Corporation, Armonk, NY, USA) [29], analyses included descriptive analyses, followed by correlational and mediation analyses. Missing data were minimal and serial means were used for replacement. Two mediation analyses, utilizing the PROCESS macro for SPSS [30], were completed to examine appearance motives and health and fitness motives for exercise as possible mediators of the relationship between (a) body surveillance and exercise avoidance motivation and between (b) body appreciation and exercise avoidance motivation. The bootstrap sample was set to 5000 for bias-corrected confidence intervals and the confidence interval was set to 95. In the first analysis, body surveillance was entered as the predictor variable, exercise avoidance motivation as the outcome, and appearance reasons and health and fitness reasons for exercise as mediators. In the second analysis, body appreciation was entered as the predictor variable. In both analyses, age, BMI, and race were entered as covariates.

## 3. Results

### 3.1. Descriptive Findings

Descriptive statistics and correlations are reported in Table 1. Hypothesis a (participants would experience some degree of exercise avoidance motivation) was supported as the majority of participants reported some level of desire to avoid exercise. Of the 131 participants, only 22 indicated that they did not feel any exercise avoidance motivation whatsoever. Hypothesis b (women’s feelings about their bodies would be associated with the extent to which they feel a desire to avoid exercise) was also supported. As expected, body surveillance was positively related to exercise avoidance motivation and body appreciation was negatively related to exercise avoidance motivation.

### 3.2. Mediational Findings

**Figure 1:**
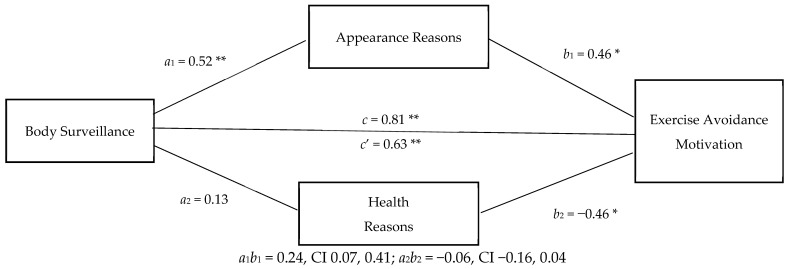
Appearance and Health Reasons for Exercise as Mediators of the Body Surveillance–Exercise Avoidance Motivation Relationship. * *p* < 0.05, ** *p* < 0.001.

Hypothesis c (reasons for exercise would mediate the relationship between body surveillance and exercise avoidance motivation) was partially supported (see Figure 1). The indirect effect of body surveillance on exercise avoidance motivation through appearance reasons for exercise, 0.24 (*SE* = 0.09), was significant (95% CI 0.07, 0.41). Health and fitness reasons for exercise did not mediate the relationship between body surveillance and exercise avoidance motivation, indirect effect −0.06 (*SE* = 0.05, 95% CI −0.16, 0.40). The direct effect of body surveillance on exercise avoidance motivation remained significant in the mediated model. Thus, appearance reasons for exercise partially mediated the association.

**Figure 2:**
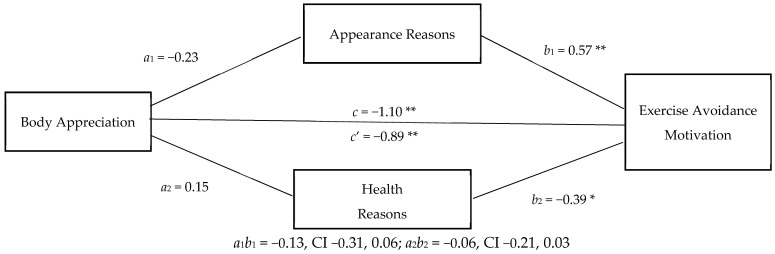
Appearance and Health Reasons for Exercise as Mediators of the Body Appreciation–Exercise Avoidance Motivation Relationship. * *p* < 0.05, ** *p* < 0.001.

In the body appreciation model (see Figure 2), hypothesis d (reasons for exercise would mediate the relationship between body appreciation and exercise avoidance motivation) was not supported. The direct effect of body appreciation on exercise avoidance motivation was significant, −0.89 (*SE* = 0.17, 95% CI −1.22, −0.56), and not mediated by either appearance reasons, indirect effect −0.13 (*SE* = 0.09, 95% CI −0.31, 0.06), or health and fitness reasons for exercise, indirect effect −0.06 (*SE* = 0.06, 95% CI −0.21, 0.03). An effect size of 0.55 was calculated using G*power post hoc analysis for both models.

## 4. Discussion

The experiences of physically active women living in larger bodies are not well understood. As expected, most of the women in our sample experienced some degree of exercise avoidance motivation, yet still engaged in activity. Understanding what factors and experiences may contribute to women’s activity despite feeling a desire to avoid it provides a starting point for future research that can inform theory and practice. Women’s feelings about their bodies were related to the degree to which they felt motivation to avoid exercise, in line with our hypothesis. Women who engaged in body surveillance had stronger motivation to avoid exercise, whereas women with higher levels of body appreciation had lower motivation to avoid exercise. These findings, while not unique, confirm previous research [3,19] and add to the knowledge base by including a sample of women living in larger bodies.

In exploring reasons for exercise as potential mediators between body surveillance, body appreciation, and exercise avoidance motivation, our results indicated only that appearance-oriented reasons for exercise partially mediated the relationship between body surveillance and exercise avoidance motivation. The current results align with the tenants of Objectification Theory [15]. In a culture that tends to objectify women [15], it is not surprising that the connection between body surveillance and exercise avoidance motivation may be intensified among women who value, and are motivated by, appearance. Women of all shapes and sizes are socialized to see the body as infinitely malleable via hard work and self-discipline [31,32]; yet the reality is that very few women are able to conform to narrow conceptualizations of the socially idealized physique. Thus, feelings of personal failure often accompany women’s experiences related to exercise and weight control [33], and may contribute to a cycle of body surveillance and motivation to avoid exercise. Research is needed to determine if and how such a cycle might function, particularly among women living in larger bodies. Evidence suggests that a focus on appearance motives for exercise should be avoided [34,35]. Although this recommendation is not novel, it does reinforce an important point; that is, that a focus on appearance within exercise and fitness settings is likely to do harm and should be avoided. This point is particularly salient given that weight loss and attaining an attractive, ‘fit’ appearance via exercise are often marketed to women, especially women who live in bodies that do not align with the thin ideal [36].

Body appreciation, on the other hand, may have potential benefits for women living in larger bodies regardless of reasons for exercise. Additional research is needed to further document this pattern of associations; however, physical activity, particularly when body appreciation is emphasized, seems to have potential for supporting and fostering physical and mental health. Greenleaf and Hauff [37] suggest that through participation in mindful physical activities, girls and women have opportunity to have agency, engage in self-care, and push back against objectification. It seems reasonable to expect that these experiences and benefits can also be attained by women living in larger bodies. As fat activists and advocates [38,39], along with leaders in weight neutral approaches to health [40], push back against dominant sociocultural beliefs about exercise for appearance and weight control, there is opportunity for messaging and practices related to body acceptance and appreciation to gain more widespread footing. Researchers and health practitioners are leading the movement toward more empowering and accepting exercise and physical activity experiences for women of all shapes and sizes [41,42].

## 5. Conclusions

Taken together, the current study findings suggest that exercise and health professionals should avoid focusing on appearance-based reasons for exercise when working with women living in larger bodies. As our results indicate, exercising for appearance reasons may strengthen the association between body surveillance, a common experience in exercise settings, and motivation to avoid exercise. Instead, creating exercise climates that facilitate body acceptance and appreciation may go a long way toward helping women of all shapes and sizes engage in activity. Indeed, our study findings indicate that body appreciation, regardless of exercise motives, is associated with less motivation to try to avoid exercise.

In interpreting the results of this study, recognizing the study limitations is useful. First, the cross-sectional design does not allow for determination of causal order. Additional research is needed to determine the temporal nature of the associations explored in the current study, as such determination could better guide future interventions and programming. Second, the participants were recruited by an online survey service (i.e., Qualtrics) and may not be fully representative of the general population. As research in this area moves forward, examining sociodemographic and cultural diversity will be important to more accurately reflect the broad range of women’s experiences. Finally, as an exploratory study, only a limited set of psychological constructs were included; future work will likely need to address a wider array of theoretically grounded constructs, such as body shame, self-compassion, and embodiment. The current work serves as a building block for future research that is needed to determine if and how de-emphasizing appearance and emphasizing health motives for exercise may contribute to a lessening of motivation to avoid exercise by offsetting the negative influence of body surveillance and strengthening the positive influence of body appreciation.

## Figures and Tables

**Table 1:** Means, standard deviations, and correlations among study variables.

Measure	M (SD)	Body Surveillance	Body Appreciation	Appearance Motives	Health Motives
Body Surveillance	3.72 (1.30)				
Body Appreciation	3.36 (1.00)	−0.60 ** (CI −0.73, −0.44)			
Appearance Motives	4.27 (1.56)	0.46 ** (CI 0.30, 0.61)	−0.17 (CI −0.22, 0.00)		
Health Motives	5.46 (1.24)	0.05 (CI −0.13, 0.22)	0.17 (CI −0.01, 0.27)	0.58 ** (CI 0.43, 0.72)	
Exercise Avoidance Motivation	4.13 (2.08)	0.50 ** (CI 0.35, 0.65)	−0.50 ** (CI −0.69, −0.39)	0.32 ** (CI 0.16, 0.49)	−0.02 (CI −0.20, 0.15)

** *p* < 0.001. CI = confidence interval.

## Data Availability

The data presented in this study are available on request from the corresponding author. The data are not publicly available due to IRB protocol.

## References

[1] Flint S.W., Reale S. (2018). Weight stigma in frequent exercisers: Overt, demeaning and condescending. J. Health Psychol..

[2] Schvey N.A., Sbrocco T., Bakalar J.L., Ress R., Barmine M., Gorlick J., Pine A., Stephens M., Tanofsky-Kraff M. (2017). The experience of weight stigma among gym members with overweight and obesity. Stigma Health.

[3] Vartanian L.R., Shaprow J.G. (2008). Effects of weight stigma on exercise motivation and behavior: A Preliminary investigation among college-aged females. J. Health Psychol..

[4] Bombak A.E. (2015). Obese persons’ physical activity experiences and motivations across weight changes: A qualitative exploratory study. BMC Public Health.

[5] Greenleaf C., Hauff C., Klos L., Serafin G. (2020). “Fat People Exercise Too!”: Perceptions and realities of shopping for women’s plus-size exercise apparel. Cloth. Text. Res. J..

[6] Meadows A., Bombak A.E. (2019). Yes, we can (No, you can’t): Weight stigma, exercise self-efficacy, and active fat identity development. Fat Stud..

[7] Rothblum E., Solovay S. (2009). The Fat Studies Reader.

[8] Sniezek T. (2019). Running while fat: How women runners experience and respond to size discrimination. Fat Stud..

[9] Strelan P., Mehaffey S.J., Tiggemann M. (2003). Self-objectification and esteem in young women: The mediating role of reasons for exercise. Sex Roles.

[10] Gilchrist J.D., Pila E., Castonguay A., Sabiston C.M., Mack D.E. (2018). Body pride and physical activity: Differential associations between fitness- and appearance-related pride in young adult Canadians. Body Image.

[11] Prichard I., Tiggemann M. (2008). Relations among exercise type, self-objectification, and body image in the fitness centre environment: The role of reasons for exercise. Psychol. Sport Exerc..

[12] Vartanian L.R., Wharton C.M., Green E.B. (2012). Appearance vs. health motives for exercise and for weight loss. Psychol. Sport Exerc..

[13] Tylka T.L., Homan K.J. (2015). Exercise motives and positive body image in physically active college women and men: Exploring an expanded acceptance model of intuitive eating. Body Image.

[14] Homan K.J., Tylka T.L. (2014). Appearance-based exercise motivation moderates the relationship between exercise frequency and positive body image. Body Image.

[15] Fredrickson B.L., Roberts T.-A. (1997). Objectification theory. Psychol. Women Q..

[16] Calogero R.M., Tantleff-Dunn S., Thompson J.K. (2011). Self-Objectification in Women: Causes, Consequences, and Counteractions.

[17] Wu Y.-K., Berry D.C. (2018). Impact of weight stigma on physiological and psychological health outcomes for overweight and obese adults: A systematic review. J. Adv. Nurs..

[18] Tylka T.L. (2019). Focusing on the Positive: An Introduction to the Volume.

[19] Cox A.E., Ullrich-French S., Tylka T.L., McMahon A.K. (2019). The roles of self-compassion, body surveillance, and body appreciation in predicting intrinsic motivation for physical activity: Cross-sectional associations, and prospective changes within a yoga context. Body Image.

[20] Oswald A., Chapman J., Wilson C. (2017). Do interoceptive awareness and interoceptive responsiveness mediate the relationship between body appreciation and intuitive eating in young women?. Appetite.

[21] Avalos L., Tylka T.L., Wood-Barcalow N. (2005). The Body Appreciation Scale: Development and psychometric evaluation. Body Image.

[22] Christel D.A., Dunn S.C.W.N. (2018). What plus-size means for plus-size women: A mixed-methods approach. Stud. Commun. Sci..

[23] McKinley N.M., Hyde J.S. (1996). The Objectified Body Consciousness Scale: Development and Validation. Psychol. Women Q..

[24] Overstreet N.M., Quinn D.M., Agocha V.B. (2010). “Beyond thinness: The influence of a curvaceous body ideal on body dissatisfaction in Black and White women”: Erratum. Sex Roles J. Res..

[25] Tylka T.L., Wood-Barcalow N.L. (2015). The Body Appreciation Scale-2: Item refinement and psychometric evaluation. Body Image.

[26] O’Neill E.A., Ramseyer Winter V., Pevehouse D. (2018). Exploring body appreciation and women’s health-related quality of life: The moderating role of age. J. Health Psychol..

[27] Cash T.F., Now P.L., Grant J.R. (1994). Why do women exercise? Factor analysis and further validation of the Reasons for Exercise Inventory. Percept. Mot. Skills.

[28] Vartanian L.R., Novak S.A. (2011). Internalized societal attitudes moderate the impact of weight stigma on avoidance of exercise. Obesity.

[29] IBM (2017). SPSS.

[30] Hayes A.F. Introduction to Mediation, Moderation, and Conditional Process Analysis. http://afhayes.com/introduction-to-mediation-moderation-and-conditional-process-analysis.html.

[31] Grogan S. (2016). Body Image: Understanding Body Dissatisfaction in Men, Women and Children.

[32] Wang X., Teng F., Chen Z., Poon K.-T. (2020). Control my appearance, control my social standing: Appearance control beliefs influence American women’s (not men’s) social mobility perception. Personal. Individ. Differ..

[33] Pila E., Solomon-Krakus S., Egelton K., Sabiston C.M. (2018). “I am a fat baby, who moved to a fat child, who moved to a fat teenager, who moved to a fat adult”: Women’s reflections of a lifetime of body and weight concern. J. Women Aging.

[34] Fuller-Tyszkiewicz M., Dias S., Krug I., Richardson B., Fassnacht D. (2018). Motive- and appearance awareness-based explanations for body (dis)satisfaction following exercise in daily life. Br. J. Health Psychol..

[35] Panão I., Carraça E.V. (2020). Effects of exercise motivations on body image and eating habits/behaviours: A systematic review. Nutr. Diet..

[36] Simpson C.C., Mazzeo S.E. (2017). Skinny is not enough: A content analysis of fitspiration on Pinterest. Health Commun..

[37] Greenleaf C., Hauff C. (2019). Environments that Cultivate Positive Embodiment Through Mindful Movement.

[38] Cooper C. (2016). Fat Activism: A Radical Social Movement.

[39] Lupton D. (2018). Fat.

[40] Souza B.J. (2015). A weight-neutral approach to health and fitness instruction. ACSMs Health Fit. J..

[41] Ebbeck V., Austin S. (2018). Burning off the fat oppression: Self-compassion exercises for personal trainers. Fat Stud..

[42] Pickett A.C., Cunningham G.B. (2017). Physical activity for every body: A model for managing weight stigma and creating body-inclusive spaces. Quest.

